# Hospital practitioner views on the benefits of continence education and best ways to provide training

**DOI:** 10.1002/nop2.1582

**Published:** 2023-01-12

**Authors:** John Percival, Katharine Abbott, Theresa Allain, Rachel Bradley, Fiona Cramp, Jenny Donovan, Candy McCabe, Kyra Neubauer, Sabi Redwood, Nikki Cotterill

**Affiliations:** ^1^ Faculty of Health and Applied Sciences University of the West of England Bristol UK; ^2^ Complex Assessment and Liaison Service North Bristol NHS Trust Bristol UK; ^3^ Medicine for Older Persons University Hospitals Bristol NHS Foundation Trust Bristol UK; ^4^ Geriatric & Orthogeriatric Medicine University Hospitals Bristol NHS Foundation Trust Bristol UK; ^5^ Bristol Medical School, University of Bristol Bristol UK; ^6^ College of Health, Science and Society University of the West of England Bristol UK

**Keywords:** catheters, continence care, education, hospital healthcare practitioner, incontinence, nurses, pads, product, training

## Abstract

**Aim:**

The aim of the study was to explore practitioners' experiences and perspectives on continence training, in order to understand its relevance to practice and how take‐up of, and engagement with, such training may be improved.

**Design:**

27 qualitative interviews were conducted with nursing, medical and allied health practitioners in three hospitals.

**Methods:**

We analysed data thematically, both manually and with the aid of NVivo software. The research adheres to the consolidated criteria for reporting qualitative research checklist.

**Results:**

Practitioners asserted the likely benefits of evidence‐based continence training, including more judicious use of products, reduction in associated infection, better patient skin care and more facilitative communication with patients. Practitioners also identified preferred methods of continence training, according to their role and workload. To ensure better take‐up of, and engagement with, continence training, it must be authorized as essential and provided in ways that reflect professional preferences and pragmatic resource considerations.

## 1 INTRODUCTION

Over 14 million UK citizens experience bladder control problems and 6.5 million suffer bowel control difficulties (NHS England, 2018); globally, the World Health Organization report that urinary incontinence is a highly prevalent condition in older people aged 60 years and over (WHO, 2017). Many who experience incontinence receive poor quality care and/or insufficient support (Abrams et al., 2017; NHS England, 2018). Continence education is key to improvements in care, but relevant training provision, particularly in the hospital setting, is often lacking or not taken up (Harari et al., 2014). Lack of continence training among healthcare professionals is a worldwide issue (International Continence Society, 2009; Read, 2018). There is a clear need for improved continence care and training, together with a better understanding of healthcare workers' perspectives, as evidence is limited in these respects. (Harari et al., 2014; Wagg et al., 2017) As a result of these concerns, our study set out to investigate the likely merits of continence training in hospitals and how education opportunities may be engaged with by more practitioners in that setting.

## 2 BACKGROUND

Studies regularly highlight the emotional, social and physical impact of incontinence on patients, including anxiety, loss of self‐esteem, decreased independence and isolation (Abrams et al., 2015; Holt‐Lunstad et al., 2015; Ramage‐Morin & Gilmour, 2013). Furthermore, incontinence substantially increases the risks of lengthy hospitalization and nursing home admission in older adults (All Party Parliamentary Group Report, 2011) These wide‐ranging effects indicate that appropriate management of incontinence symptoms is therefore essential and pro‐active continence care can make demonstrable improvements (Abrams et al., 2017; NHS England, 2014; Wagg et al., 2017).

Treatment of incontinence, however, has often been reported as inadequate (Healthcare Quality Improvement Partnership, 2010; Taylor & Cahill, 2018), particularly for older adults (Harari et al., 2014; Vethanayagam et al., 2017). Indeed, the Francis public inquiry reported that the quality of continence care was the area of practice most frequently subject to complaint (Francis, 2013). Falls, pressure ulcers and moisture lesions are directly linked to poor continence care in older adults (DoH, 2014; Yates, 2017). Studies have also found that healthcare professionals in acute care settings inconsistently identify, assess and conservatively manage incontinence, citing evidence of inappropriate use of catheters and pads, weak care planning and insufficient staff support and training (Age UK et al., 2018; Orrell et al., 2013; Wagg et al., 2008).

Practitioner education is recognized as a crucial component of moves to improve the quality of continence care (All Party Parliamentary Group Report, 2011; NICE, 2019; Orrell et al., 2013). However, the availability of continence care training, and healthcare practitioners' engagement with it, are reported to be limited (Harari et al., 2014; United Kingdom Continence Society, 2015). There has been little exploration of why this is the case, beyond noting that continence care is sometimes seen by practitioners as unglamorous and a sensitive subject to discuss with patients (Read, 2018). A recent paper, also based on the study reported here, found that hospital practitioners believe continence care can be improved in patient‐centred, time‐efficient ways, if they had appropriate continence training (Percival et al., 2021). In this current paper, we closely examine hospital practitioners' experiences of relevant training, their perspectives on its benefits to practice, and their preferences for the ways in which it is delivered, so as to understand how continence education can best be provided, taken up and engaged with by more staff in the hospital setting.

## 3 THE STUDY

### 3.1 Aims

The study aspired to understand hospital healthcare practitioners' views on the efficacy of continence education and the ways in which take‐up and engagement with continence training can be improved.

### 3.2 Design

The interview schedule was not formally piloted but informed by previous audit data and existing evidence (Bowling, 2014), as well as by consensus input by experts in the clinical area, and reviewed by all authors. The interview schedule (see Appendix 1) included questions on how participants had learned about continence care, whether continence training had been made available, whether participants knew how to access such training, preferences regarding style of training and participants' awareness of continence care guidelines. As such, the study design was deliberately exploratory, offering opportunity for participants to reflect on relevant experiences and ideas about a health topic that has received relatively little attention (Hunter et al., 2018).

### 3.3 Participants

Research participants were recruited in acute inpatient wards (mostly, though not exclusively, care of older people wards) at three hospitals in England: two large, city, teaching hospitals (one, a tertiary centre for Urology) and one a smaller, town‐based non‐teaching centre. The main inclusion criterion was that research participants have responsibility for patients aged 65 and over. Twenty‐seven participants were recruited, including nursing staff, allied healthcare professionals, medical staff and healthcare assistants. All participants answered the questions put to them.

Recruitment was carried out through purposive sampling of staff groups to achieve representation across professional roles (Bowling, 2014). The lead researcher (JP) met with ward managers at each hospital to discuss the research and provide participant information materials for display and distribution among staff. Once representation was achieved in respect of each staff group, nursing, medical and allied health, the sample size was determined during ongoing analysis and interpretation of emerging themes, ensuring data collection parameters in line with the study's objectives (Johnson et al., 2020). One‐off Individual interviews took place in the hospital settings and were carried out by JP.

### 3.4 Data collection

Semi‐structured exploratory interviews were carried out with hospital‐based nursing, medical and allied health professional staff, to probe perspectives on continence care practice and ways to optimize such care. Interviews were audio‐recorded, transcribed verbatim and then analysed using thematic analysis (Silverman, 2015). Initial data analysis involved close and repeated reading of each transcript by JP, establishing an opportunity for the development and refining of coding strategies in relation to emerging issues and insights. Transcript data were then imported into the qualitative data management software package NVivo 12 (King, 2004) with coding further enhanced by JP and the chief investigator (NC) using a coding frame devised to highlight theme connectivity and relevance to the primary areas of investigation (Silverman, 2015).

### 3.5 Ethics

All interviews with healthcare practitioners were carried out in accordance with research governance ethics protocols and Health Research Authority (HRA) approval (Research Ethics Committee reference: HAS.19.07.221, September 2019). Written consent was provided by each participant prior to interview.

### 3.6 Rigour

In order to build trustworthiness and quality about data analysis, the conduct and thematic outcomes of analysis were considered and agreed on by all authors. Furthermore, regular meetings of the researchers took place to discuss emerging themes and ratify their origin in the data. In addition, researcher reflexivity and peer review regularly took place during stages of data gathering. The research adheres to the COREQ reporting checklist (Tong et al., 2007).

## 4 RESULTS

The study's total of 27 participants was evenly distributed across the three hospital sites, as shown in Table 1. This total included 17 nursing staff, 5 allied healthcare professionals, 3 medical staff and 2 healthcare assistants. Participants represented all age groups, were mainly female (as is usual in the healthcare workforce) and were predominantly white British, reflecting the regional demographic.

**TABLE 1:** Hospital healthcare practitioner characteristics

Setting	Prof title	Age band	Gender	Practice years	Band	Ethnicity
A	Staff nurse	21–25	F	1.5	5	Black British
A	Staff nurse	21–25	F	<1	5	White & Black Caribbean
A	Ward sister	26–30	F	6	7	White British
A	Senior staff nurse	26–30	F	6	6	White British
A	Senior staff nurse	51–55	F	9	6	White British
A	Senior staff nurse	26–30	F	5	6	White British
A	Apprentice healthcare support worker	18–20	F	<1	2	White British
A	Therapy technician	31–35	F	6.5	4	White British
A	Senior nursing assistant	46–50	F	13	3	White British
B	Staff nurse	56–60	M	29	5	White British
B	Staff nurse	56–60	F	40	5	White British
B	Student nurse	31–35	F	<1	N/A	White British
B	Physiotherapist	21–25	F	1.5	5	White British
B	Therapy technician	46–50	F	6	3	White British
B	Consultant physician	36–40	M	14	N/A	Indian
B	Nursing assistant	36–40	F	2	2	White British
B	Staff nurse	46–50	F	2	5	White British
B	Junior sister	31–35	F	9	6	White British
C	Sister	21–25	F	4	6	White British
C	Assistant nursing practitioner	31–35	F	5	4	Black South African
C	Staff nurse	26–30	F	1	5	White British
C	Consultant geriatrician	36–40	M	17	N/A	White British
C	Physiotherapist	41–45	F	15	6	White British
C	Dementia specialist practitioner	51–55	F	35	7	White British
C	Occupational therapist	41–45	F	11	6	White British
C	Healthcare assistant	51–55	F	3	3	White British
C	Junior doctor	21–25	F	<1	F1	White British

*Note*: Table shows participants' setting (anonymized), role, age range, gender, length of time in practice, salary band where appropriate and ethnicity.

In order to safeguard confidentiality, the source of interview excerpts included in this paper is identified using a letter signifying each hospital site, as shown in Table 1, followed by the practitioner's study identification number.

The questions on training put to practitioners elicited opinions and experiences regarding four themes: challenges to understanding and managing continence care; availability of continence care education; perceived benefits of continence care education; and preferred methods of training.

### 4.1 Challenges to understanding, monitoring and managing continence care needs

Although incontinence was rarely the principal reason for a patient's admission, practitioners' accounts suggested that it featured prominently in their everyday practice. Certainly, staff on ‘care of elderly’ wards across the three sites refer to numbers as high as 75–80 per cent of patients who “struggle in some way with their urinary or faecal output” [A02, apprentice healthcare support worker], leading one consultant physician to describe incontinence as “one of the geriatric giants” [B46].

Despite the prevalence of older hospital patients with incontinence, it was clear from interviews across the three sites that pro‐forma hospital admission assessment procedures insufficiently captured continence care needs, due to “variable” or sometimes “incomplete” detail. Accurate record‐keeping by ward staff, following routine checks of patients' skin condition, temperature and urine/faecal output, was therefore seen as of compensatory importance to building a better understanding of the patient's continence care needs. However, participants disclosed that monitoring and record‐keeping were not always carried out efficiently:

This morning a lady was TWOC'd [attempted a Trial WithOut Catheter]. she is mobile, gone to the toilet a couple of times last night but nobody has documented to see if she has passed urine or not or how much. [A04; senior nursing assistant]

Lack of oversight and erroneous assumptions could result in patients using inappropriate products that jeopardized independence:

I have seen situations where people have been put in incontinence pads and then you ask them or you ask their relative and [are told] they do not use them at home. And it's kind of, I think we sometimes get in the habit of presuming that somebody needs that when they do not. [C23, staff nurse]

A ward sister observed that lack of monitoring regarding products such as catheters showed how continence care was sometimes not “thought through,” with the result that aspects of conservative management, such as dietary advice, bladder retraining or pelvic floor muscle exercises, were not adequately considered. A nursing assistant suggested that lack of training had a particular bearing on this issue:

When [staff]… have just been in the profession for a long time, pads just go on [patients]… A lot of people have not had training and just see incontinence pads and they are almost saying, “[I use them] just in case.” When really we should be moving away from that. [B47, nursing assistant].

The relevance of continence care training to meeting the challenges outlined above was apparent throughout interviews. The availability of training made available to practitioners was therefore explored further.

### 4.2 Availability of continence care education

The majority of nursing staff interviewees told us they had received no “specific,” “formal,” hospital‐based training in continence care, not even as nursing assistants, but had learned about it from industry representatives or simply by “just doing it,” learning “on the job”:

We have had people come in and talk to us about incontinence products like different types of catheters and different pads but not any specific training about incontinence as a whole. [C21, staff nurse]

I have been here six‐and‐a‐half years and I have never had any official continence, incontinence teaching. [A03, therapy technician]

Nursing staff referred to study days that had been organized on “pain,” “mouth care” or other aspects of fundamental practice, with nothing comparable in respect of continence care. A small number of interviewees recalled incontinence being a noteworthy component of their student education and a few said their Hospital Trust “induction” had included a session on the management of patients with incontinence; otherwise, interviewees had gained knowledge through self‐directed reading, mostly online. Staff indicated that although online learning had been useful, they were more likely to engage with training on continence care, and its complexities, if it was presented in person by knowledgeable and experienced practitioners.

This lack of concerted continence training was a concern for many interviewees. Some spoke of the pressure on new staff, who may have “no background in care” [C22, nurse assistant], or the pressure on mental health nurses who, according to a junior doctor, “are not ‘physically’ trained nurses” but often deal with incontinence issues [C29]. Healthcare assistants, too, were said to be disadvantaged, given that they work with “high continence needs but haven't really had much training in it” and so may be unaware, for example, of “the link between skin [integrity] and continence” [C23]. Doctors also conceded “we get very little [continence care] training… it's not high enough on our curriculum” [C24], and “if we did have [continence care] training we might be able to stop these situations where we have forgotten to TWOC the patient” [C29].

A minority of practitioners referred to occasional episodes of training on aspects of care connected with incontinence, such as skin care. Some practitioners referred to industry “reps” who came on to the ward to provide updates on products, such as new incontinence pads and their correct use, or catheters and new discharge packs. Although most interviewees said they were aware of good practice guidelines on continence care in hospitals, few confirmed they had actually read them or been asked to do so. A number of interviewees referred to the availability of urology nurses, or senior staff, should they need advice on a continence care issue. However, none of our interviewees knew of, or any longer had access to, a designated, specialist, continence care advisor in their hospital. According to one ward sister, the lack of specialist advice contributes to “poor” continence care practice.

**FIGURE 1:**
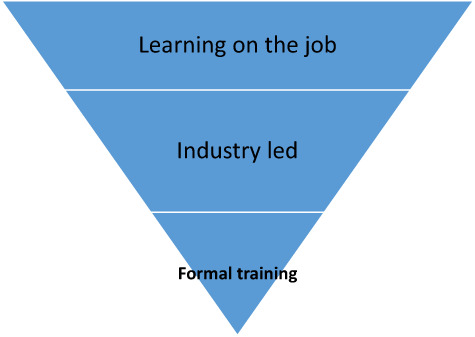
Sources of education about continence care in decreasing proportions.

Given these experiences of training availability, Figure 1, below, is presented to graphically depict the lesser availability of formal training in comparison with other sources of continence knowledge. Sources of education are shown in decreasing proportions, as described by practitioners.

Practitioners suggested that dedicated continence care education would benefit their practice by getting people “thinking” more consciously about the subject, so “it would be at the forefront of our mind more” [A05, staff nurse]. Similarly, a senior staff nurse advocated raising the profile of continence care training when she said:

We are very much aware of dementia, we are very much aware of Parkinson's. I think continence needs to be brought up to the forefront as well. [A09, senior staff nurse]

### 4.3 Perceived benefits of continence care education

Practitioners' accounts indicate that continence care education would help improve their “management of incontinence” [C21] and provide much needed information about “how we manage it without going for the easy option [automatic use of pads and catheters]” [A05]. The management and support of patients in respect of their skin integrity was a particular concern for practitioners, as the following excerpt exemplifies:

We are constantly managing patients who have really poor skin from continence issues. I do not really know myself if there are other things we could be doing… We do our best but if we had kind of more education and understanding then maybe our care would be better. [C23, staff nurse]

In this connection, practitioners predicted that continence care education could play a part in helping staff “get rid of” incontinence sheets, which were in some cases still used inappropriately and “causes their skin to break down” [A01]. Practitioners also believed that continence care education would help improve their knowledge of relevant best practice guidelines, “what we should be doing, what is sort of the baseline that we should all be meeting for our patients” [B47].

According to practitioners, improved access to relevant education would also facilitate more efficacious use of incontinence pads:

I think there needs to be training to avoid seeing continence just in terms of incontinence and needing a pad. [C28, occupational therapist]

Practitioners repeatedly said that continence training would help ensure safe use of continence care products and “training with awareness of why we are putting a pad on” [C27] as well as guidance on “how to make sure [staff] have got the right size pad” [C29] and “how often you change pads… we don't know” [B42]. As regards catheters, one therapy technician suggested staff would benefit from “teaching around… weighing up the effects of long term catheter and short term catheter [use]” [A03]. Provision of such teaching, it was said, would increase staff vigilance and caution regarding catheter use.

In addition, practitioners spoke of the potential impact of training in respect of increasing their knowledge of local community continence care services and sources of “community support” [A03], to facilitate discharge planning and “signpost [patients] in the right way” [A08]. This better co‐ordination of continence care was thought likely to further raise its profile and also help support patients' independence:

I think staff knowledge [needs improving]… with knowledge we can then give patients the confidence that they can manage their own continence, whether its catheter, pads, whatever. [B45, therapy technician]

A number of practitioners also advocated more attentive listening to patients and their family members, in order to “get their perspectives” in regard to continence care and planning. In this context, a junior ward sister acknowledged that staff had to be sufficiently confident “to ask those questions and not everyone is confident to do that” [B49], a situation that prompted participants to reflect on communication shortcomings:

You can go over to somebody and say its fine, do not worry. When actually they are like, “I am worrying and it's not fine, it's not nice.” Sometimes it would maybe be nice to know how they would want us to approach it as opposed to [us] just batting it off. [A08, senior staff nurse]

For all the reasons outlined above, participants often spoke of continence care training with conviction and in the spirit of broadening professional horizons:

I would love to have some [continence care] training… we had extra [mouth care] training and I learnt things that I never knew about mouth care… without that extra training, sometimes you do not get to know. So I think we could all learn something new [about continence care] if we just got given a little bit of extra training. [B49, junior ward sister]

What form this potential training should adopt was a question we put to practitioners, in order to develop our understanding of provision that takes account of needs and preferences and is, therefore, likely to be approved and supported.

### 4.4 Preferred methods of training

When asked to consider their preferences regarding methods of training, opinions were mixed as to the advantages of regular, short duration, ward based, sessions or less frequent, half/full day, off‐site, study sessions. No practitioner elected online e‐learning as a preferred source of training. Generally, nursing staff favoured shorter, regular, “hands‐on” training sessions and medical and allied health professionals intimated a preference for longer, occasional workshop‐type provision. It is worth briefly setting out the reasons given by interviewees for their preferences, as these provide further insight into the likely interest in, and take up of, continence care training for hospital‐based staff.

Staff speaking in favour of regular, short duration, ward‐based training said they normally found it easier to focus and retain information with this format and, additionally, it offered opportunity for hands‐on, practice‐oriented learning:

We do have this teaching scheme where different people will come in and talk about different things… every week… it's short and sweet, I think that seems to work well. [A01, senior staff nurse]

This format was also said to be practical, given the pressures on staff time and the difficulty releasing staff for lengthier periods of off‐site training. A staff nurse amplified this last point when she said:

Little short bursts [of training] happening on the ward area I think is ideal. Because everybody is getting that same opportunity. Because study days, you can only release so many people at a time, if you are lucky. [A09, senior staff nurse]

In addition, the advantage of “localised training coming in” was mentioned by practitioners who welcomed gaining greater knowledge, in this way, of relevant continence services that support people at home, enabling them to advise patients of “what is available [in the community] for people that are incontinent” [B45, therapy technician].

Lengthier, off‐site, training workshops was a format favoured by one consultant [C24] as a more practical option for medical staff in hospitals, given ward‐based demands on doctors:

For doctors, one‐day training works well because the chance of getting us back to [short, follow‐up] things is low. For nurses I think it's the other way round. In the past when I have tried to do training for nurses it's almost impossible for them because they cannot get off the wards. [C24, consultant geriatrician]

Another consultant endorsed this view and added that he preferred this workshop‐type training as it offered sufficient time for audience participation and the generation, by speakers, of ideas and “insights.” A relatively small number of nursing and therapy staff elected the off‐site, workshop training option, on the grounds that it offered the opportunity to hear from a number of different speakers and practitioners about various services and aspects of practice, “away from the practice area [where] you're not being pulled in all directions” [C26; dementia specialist practitioner]. One or two interviewees were open to “mixed methods” training. A physiotherapist said she likes power‐point presentations supplemented by “learning through doing, otherwise it doesn't stick” [B44].

Whatever the format, regularity of training was advocated as entirely appropriate, to ensure all staff are included regardless of shift patterns. One ward sister cautioned, however, that time pressures may make commitment to any format of training vulnerable to disruption and could only be guaranteed to take place if authorized as “essential training” [A07, ward sister].

## 5 DISCUSSION

Our research findings highlight three key topics that merit discussion: adequacy of the continence care training available to hospital healthcare practitioners; the benefits to continence care practice of an increase in practitioner education; and ways in which continence care training might be promoted and delivered to maximize take‐up by practitioners. Our study finds that relevant training is negligible, that practitioners want more continence education and that they have clear ideas on how such education can best be provided.

Studies have drawn attention to the availability of formal continence care training to healthcare practitioners and its occurrence in less than half of acute hospitals (United Kingdom Continence Society, 2015). Our study bears out these concerns and indicates the relative prominence of industry‐led, product‐focused instruction and the lack of service‐led, broad, evidence‐based training. Industry‐led training, we suggest, may exacerbate practitioners' over‐reliance on catheters and pads, while independent, comprehensive training is less influenced by marketing and likely to help practitioners question, and learn more from, their practice. Provision of training driving continence promotion is key.

Previous survey findings have also exposed weaknesses in the quality of student continence education. A survey of undergraduate programmes including medicine, nursing, physiotherapy and occupational therapy, in 84 UK universities, found significant lack of time allocated to continence education (an average of 7.3 hours for student nurses, 4.9 hours for medical students, 3.8 hours for student physiotherapists and 3.5 hours for occupational therapy students). The survey concluded that continence education was insufficiently supported as a stand‐alone topic to approved standards within undergraduate curricula (McClurg et al., 2013). Studies have found evidence for continence education to be given greater emphasis in student training and for its content to be more fully developed (Ferdinand, 2018; Gourlay, 2011; Holroyd, 2015).

Of relevance here are the recently updated standards of proficiency for Registered Nurses, which now stipulates that practitioners should use evidence‐based approaches to meet continence needs (Nursing and Midwifery Council, 2018). In the literature search carried out for our study, there was no similar reference to continence education in the proficiency protocols pertaining to medical or allied health practitioner registration. Furthermore, over half of qualified nurses do not receive any post‐registration continence education (Ferdinand, 2018).

Increased continence care education helps build a more knowledgeable workforce, capable of routinely carrying out timely, person‐centred assessments, enabling greater patient self‐management and more cost‐effective treatment (Holroyd, 2015; Nursing and Midwifery Council, 2018). Indeed, relevant training would help raise healthcare practitioners' awareness and consideration of alternatives to, or more restricted use of, catheters and incontinence pads, yielding health and social care cost savings and improvements to patients and carer quality of life (Dealey et al., 2012; Murphy et al., 2015; Unplanned Admissions Consensus Committee, 2019). Our study has revealed the pertinence of such benefits to current continence care practice in hospital settings, which practitioners have told us is sometimes characterized by limited understanding, or awareness, of best practice regarding continence management, skin integrity issues and judicious use of products.

Additionally, findings from our study show that practitioners see the benefit of training that would assist them in confidently facilitating conversations with patients, to help patients overcome embarrassment and more readily impart relevant information and experience. Training that achieves these outcomes is important when providing intimate care (Redwood et al., 2020) and is highly valued by patients, who are reassured when healthcare practitioners acknowledge that incontinence is not easy to talk about and encourage a shared approach to care planning (Brett, 2021).

Recommendations have been made for continence education to be provided in ways that offer more structure, for example through modular delivery. Furthermore, the provision of continence education in different formats is key to ensuring that training is accessible, given the range of staff involved and the constraints on their time (United Kingdom Continence Society, 2015). Our study identified staff interest in various options for delivery of face‐to‐face training; in addition, education providers should consider e‐learning packages to supplement in‐person continence education sessions (Gourlay, 2011; McClurg et al., 2013). Whatever format of training delivery, practitioners need to be released from other work pressures and to have sufficient, dedicated time made available, for continence education to be viable and effective (Ferdinand, 2018).

Continence education should also feature in continuous professional development for post‐graduates if they care for patients with continence care needs (Gourlay, 2011; United Kingdom Continence Society, 2015). However, for designated training to be recognized as a necessary component within healthcare curricula, continence care has to assume a higher status and profile (Harari et al., 2014; Ferdinand, 2018). In this respect, and as our study infers, an increase in continence care leads and specialist nurses would play a significant role in raising awareness of good practice and organising and developing relevant training (Harari et al., 2014; Unplanned Admissions Consensus Committee, 2019).

To help ensure continence care training gains traction and becomes routine, there are recommendations for education programmes to be ongoing and mandatory, helping cement its status as required and necessary learning (NHS England, 2018; Orrell et al., 2013). Indeed, a recent report highlighting the plight of pelvic floor services recommends that nursing and medical education should include a sufficient emphasis on continence throughout the healthcare professional's career (The Pelvic Floor Society, 2021). Our study adds weight to this argument, given participants' view that continence care training would have to be authorized as essential if it is to take place at all.

### 5.1 Strengths and limitations

Key strengths of the study include its targeted focus on continence care training, a reportedly important but under‐researched aspect of healthcare. Practitioners' preferences for regular ward‐based training or off‐site workshops help inform continence training that is fit for purpose and with improved take‐up by staff. In addition, the data we gathered helps strengthen the health policy case to reduce the unnecessary use of products known to have adverse health consequences. A limitation of the study is an imbalance in research participants, given the higher representation of nursing staff in relation to medical and allied health practitioners. A more even spread of practitioners would potentially have helped further refine interpretations of our research data.

## 6 CONCLUSION

Obstacles to good continence care include insufficient understanding and monitoring of patients' continence needs, leading to over‐reliance on, or inappropriate use of, catheters and pads. However, the availability of broad, structured training that might address such issues is negligible and most practitioners also lack recourse to specialist advice, other than product‐focused training by industry reps. Findings indicate that concerted continence care training would help improve practitioners' management and communication of continence issues and develop safer practice. The delivery style of training needs to be flexible to reflect different practitioner preferences and pragmatic resource considerations. Most importantly, the study's findings offer insights that should help health service and training providers engage more practitioners in continence education experienced as accessible, worthwhile and effective.

## FUNDING INFORMATION

This study was supported by University Hospitals Bristol and Weston NHS Foundation Trust, Research Capability Funding (RCF) stream [grant number 2018‐Aut‐03].

## CONFLICT OF INTEREST

The authors declare that there is no conflict of interest.

## Data Availability

Data available on request due to privacy/ethical restrictions.

## APPENDIX 1 PERSPECTIVES ON CONTINENCE CARE

- How have you learned about continence care?
  ◦ Initial training for your role? Clinical placements during training? Courses since qualifying? Specialist training?
- Has continence education been available for you?
  ◦ Training: theoretical and during placement, post‐training.
- Do you know where to access continence training?
  ◦ Is this something you would want to access or other priorities?
- How do you prefer to learn?
  ◦ Online, in‐person, reading, role‐play, mixed methods. Single hit or course over extended period. Short segments or get it all done.
- What training has worked well in the hospital training and why?
  ◦ A training session that sticks in the mind, what worked well about it?
  ◦ A training session that did not go well and why?
- Awareness of continence guidelines/documents?
  ◦ Awareness of the NICE guidelines, Excellence in Continence Care guidelines?
